# Enhancing Detection of SSMVEP Induced by Action Observation Stimuli Based on Task-Related Component Analysis

**DOI:** 10.3390/s21165269

**Published:** 2021-08-04

**Authors:** Xin Zhang, Wensheng Hou, Xiaoying Wu, Lin Chen, Ning Jiang

**Affiliations:** 1 Bioengineering College, Chongqing University, Chongqing 400044, China; wuxiaoying69@163.com (X.W.); clxyz@cqu.edu.cn (L.C.); 2 Key Laboratory of Biorheological Science and Technology, Ministry of Education, Bioengineering College, Chongqing University, Chongqing 400044, China; 3 The Department of Systems Design Engineering, University of Waterloo, Waterloo, ON N2L3G1, Canada; ning.jiang@uwaterloo.ca

**Keywords:** brain-computer interface (BCI), steady-state motion visual evoked potential (SSMVEP), action observation (AO), task-related component analysis (TRCA), rehabilitation

## Abstract

Action observation (AO)-based brain-computer interface (BCI) is an important technology in stroke rehabilitation training. It has the advantage of simultaneously inducing steady-state motion visual evoked potential (SSMVEP) and activating sensorimotor rhythm. Moreover, SSMVEP could be utilized to perform classification. However, SSMVEP is composed of complex modulation frequencies. Traditional canonical correlation analysis (CCA) suffers from poor recognition performance in identifying those modulation frequencies at short stimulus duration. To address this issue, task-related component analysis (TRCA) was utilized to deal with SSMVEP for the first time. An interesting phenomenon was found: different modulated frequencies in SSMVEP distributed in different task-related components. On this basis, a multi-component TRCA method was proposed. All the significant task-related components were utilized to construct multiple spatial filters to enhance the detection of SSMVEP. Further, a combination of TRCA and CCA was proposed to utilize both advantages. Results showed that the accuracies using the proposed methods were significant higher than that using CCA at all window lengths and significantly higher than that using ensemble-TRCA at short window lengths (≤2 s). Therefore, the proposed methods further validate the induced modulation frequencies and will speed up the application of the AO-based BCI in rehabilitation.

## 1. Introduction

Action observation (AO), which can evoke the mirror neuron system (MNS), is an alternative approach for stroke rehabilitation [1]. Acting via MNS, AO can subconsciously activate the motor neurons that are responsible for producing the observed action [2]. In addition, AO has been reported to have a positive impact on stroke patients [3]. 

While AO provides involuntary sensory stimulation for patients, the effect on the brain plasticity is limited. Sensory stimulation with the user’s own volitions can further promote brain plasticity [4]. Therefore, obtaining the user’s volitions is critical. Brain-computer interface (BCI) is a novel method that can obtain individuals’ volitions to interact with external environments without using regular peripheral nerves and muscles [5]. It reveals great potentials for enhancing brain plasticity in rehabilitation, such as motor imagery (MI)-based BCI [6]. 

However, recent research has shown that some patients experience full or partial loss of MI ability following a stroke [7]. For this group of patients, Ku et al. [8] proposed a BCI-based AO, using flickering action video, to activate MNS. Mental status and feedback were determined by detecting steady-state visual evoked potential (SSVEP). However, SSVEP was induced by the flicker in the background of the video in Ku’s study. The detection did not completely reflect the engagement in the action. In our recent study [9], a gaiting stimulus was proposed. The results showed that observing the designed gaiting stimulus could simultaneously induce steady-state motion visual evoked potential (SSMVEP) in the occipital area and activate sensorimotor rhythm (SMR) in the primary sensorimotor area. No flicker existed in the stimulus and the SSMVEP was induced by the movement of the feet. However, the SSMVEP induced by the gaiting stimulus was composed of complex modulation frequencies. Those frequencies were modulated between frame rate and stride frequency. Traditional canonical correlation analysis (CCA) method suffered from poor recognition performance in identifying those modulated frequencies, especially at short stimulus durations. The average classification accuracy was only 34.8% at 1 s window length in a four-class scenario in AO-based BCI [9].

SSMVEP, which can be induced by Newton’s rings, was first proposed in [10]. Then Yan et al. [11] demonstrated that visual stimulus with periodic motion such as swing and rotation can also induce SSMVEP. SSMVEP showed a slight difference from SSVEP, e.g., the SSMVEP contained fewer harmonic components. Our recent study further explored the difference between SSVEP and SSMVEP which were induced by the stimuli containing multi-frequencies [12]. The results showed that there were differences in the components of the induced modulated frequencies. Furthermore, for the SSMVEP induced by the AO stimulus (e.g., a gaiting stimulus), the main induced frequency was at the frame rate. The sum and difference between the frame rate and twice stride frequency could also be found as shown in [9]. However, the amplitudes of these induced frequencies varied greatly, a fact which showed a great difference from SSVEP. In spite of this, the target identification methods for SSMVEP were the same as SSVEP in the existing studies.

To improve the performance of BCI, hybrid BCI and improving algorithms are two traditional approaches. Mannan et al. showed that combining eye tracking with EEG can highly improve the decoding accuracy of SSVEP-based BCIs [13]. For the algorithms in SSVEP-based BCIs, power spectrum density analysis (PSDA) was used to detect target frequency from single-channel EEG [14]. In addition, CCA was employed to improve classification accuracy by utilizing multi-channel EEGs [15]. By computing canonical correlation between the multi-channel EEG signals and the designed reference signals, the target can be identified by the largest similarity. Traditionally, the reference signals are constructed from sine-cosine signals with flash frequencies. However, such designed reference signals cannot reflect the subject-specific features [16]. Thus, individual calibration data have been incorporated in CCA-based SSVEP detection [17,18]. In addition, considering the harmonic components in SSVEPs, a filter bank CCA was proposed to decompose SSVEPs into sub-band components to extract the independent information more efficiently [19]. Recently, task-related component analysis (TRCA) was employed to improve SNR of SSVEP by removing the background EEG activities. The eigenvectors corresponding to the largest eigenvalue were selected to construct the spatial filter [20]. In addition, an ensemble method e-TRCA was developed for a high-speed brain speller [20]. This method achieved the best performance among the existing CCA-based methods. However, few studies focused on the target identification methods for the SSMVEP-based BCIs, especially for SSMVEP induced by the AO stimulus, which had great potential in BCI-based rehabilitation. Currently, CCA is still the main identification method for SSMVEP. The reference signals were constructed from sine-cosine signals containing all the induced frequencies [9,11,21]. And it required manual optimization of reference signals to achieve high identification accuracy.

In this study, TRCA was selected as the data-driven approach to construct the spatial filter for the AO-based BCI for the first time. In addition, a multi-component task-related component analysis (mc-TRCA) method was proposed to enhancing the target detection accuracy of SSMVEP induced by the AO stimuli. The performance was evaluated on the EEG data when participants gazed at the gaiting stimulus. Unlike only one significant eigenvector in SSVEP, multiple significant eigenvectors were found in SSMVEP, induced by the gaiting stimulus. Thus, the proposed method automatically selected all the significant task-related components to construct multiple spatial filters to increase the signal to noise ratio at different modulated frequencies. Then, a combination of TRCA and CCA (CB-mc-TRCA) was proposed to utilize both advantages. Finally, the results were compared with traditional CCA-based method and existing e-TRCA method. The goal of this study is to further analyze the characteristic of the SSMVEPs induced by the gaiting stimulus and to demonstrate the enhancement of the TRCA-based methods in the detection of those SSMVEPs.

## 2. Materials and Methods

### 2.1. Data Description

The proposed methods were evaluated on the dataset reported in our recent study [9].

#### 2.1.1. Stimulus

The stimulus was a novel frame-based gaiting stimulus [9], i.e., a gaiting sequence of a human as shown in Figure S1. No flicker existed in the gaiting stimulus. Our recent study [9] illustrated that observing such gaiting stimulus could simultaneously induce SSMVEP in the occipital area and activate SMR in the sensorimotor area.

The gaiting stimulus used in obtaining the EEG data consisted of four targets with different stride frequencies. The target stimuli were presented on a LCD monitor (60 Hz refresh rate) with stride frequencies *f* ($f_{1},\;f_{2},\;f_{3},\;\mathrm{and}\;f_{4}$) of 0.536 Hz, 0.75 Hz, 0.625 Hz, and 0.938 Hz in the left, right, up, and down position of the monitor, respectively. The frame rates of these four stimuli *F* ($F_{1},\;F_{2},\;F_{3},\;\mathrm{and}\;F_{4}$) were 8.57 Hz, 12 Hz, 10 Hz, and 15 Hz, respectively.

#### 2.1.2. Experiment and EEG data acquisition 

The experiment consisted of four runs with each run containing 20 trials for all the ten participants. Each trial consisted of three phases: cue phase (−2 s–0 s), stimulus phase (0 s–6 s), and relaxation phase (6 s–10 s) as shown in Figure S2. Each trial started with the cue phase, where four cue letters would appear on the screen. Then, the four gaiting stimuli would replace the cue letters, appearing for 6 s. The participant engaged his or her gaze at one target. After the stimulus phase, the detection result using CCA would be displayed in the middle of the screen and participant could relax the gaze for 4 s. In one run, each target was repeated five times. Since the experiment consisted of four runs, each target frequency contained twenty instances. All EEG data and event timestamps (the beginning and end of each trial) were recorded for subsequent processing. 

Ten healthy subjects participated in the experiment. EEG signals were recorded with a commercial research-grade EEG system (gUSBamp and Ladybird electrodes, g.tec Guger Technologies, Austria). EEG signals were recorded with 16 channels, sampled at 1200 Hz. The left earlobe was used as the reference and Fpz was used as ground. A notch filter from 58 Hz to 62 Hz was used to eliminate the power line interface.

#### 2.1.3. Data Preprocessing

The EEG data from six electrodes (O1, Oz, O2, PO3, POz, and PO4) distributed in occipital area were used for the evaluation in this study. Data points (stimulus phase: 6 s) were extracted according the recorded event. Considering the latency of 0.14 s caused by the visual pathway [22], the EEG data from 0.14 s to *τ* s were extracted, where ‘*τ*’ represented the time window used in the investigation. Then, a band-pass filter between 4 Hz and 50 Hz was applied to the EEG data. The filter was designed as a Butterworth infinite impulse response (IIR) filter of order four.

### 2.2. Multi-Components Task-Related Component Analysis

TRCA [23] was firstly proposed to maximize the reproducibility during task periods from near-infrared spectroscopy data. Since it had the ability to maximize inter-block covariance and remove the task-unrelated artifacts, TRCA was successfully used as a spatial filter to remove background EEG activities in SSVEP-based BCIs [20].

**Figure 1:**
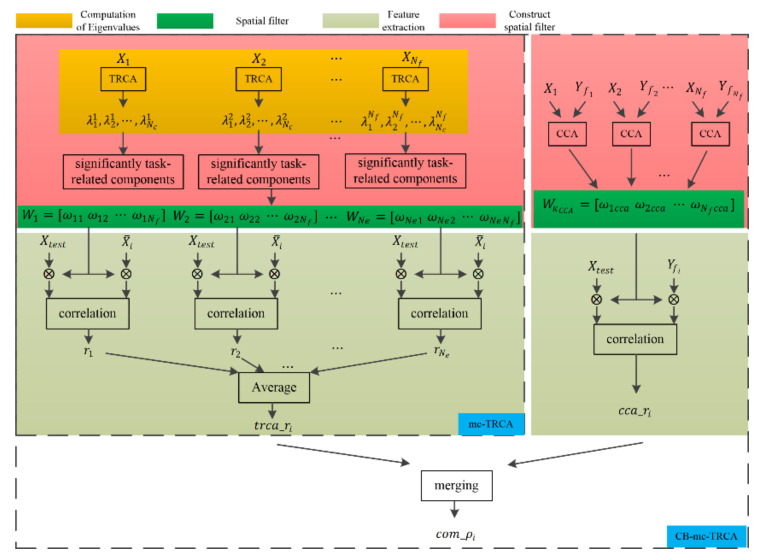
The diagram of the mc-TRCA and CB-mc-TRCA.

Unlike existing SSVEP-based studies that only selected the eigenvectors corresponding to the largest eigenvalue to construct the spatial filter, the EEG response to gaiting stimuli showed multiple task-related components (details in Section 3.1). Thus, multi-component task-related component analysis was proposed in the current study to automatically select significant task-related components in the SSMVEP induced by the gaiting stimuli. Moreover, TRCA was capable of maximizing the covariance of inter-trial SSVEP data, and the traditional CCA-based method was able to maximize the covariance between sine-cosine reference signals and SSVEP data; both TRCA-extracted features and CCA-extracted features were combined in our study. This approach may combine the advantages of CCA and TRCA. Figure 1 shows the diagram of the mc-TRCA method and CB-mc-TRCA method. The mc-TRCA method contains three parts: computation of eigenvalues, choice of significant components, and feature extraction. After computing the eigenvalues and statistical test, the spatial filter can be constructed. The details of each part are described as follows.

#### 2.2.1. Computation of Eigenvalues

Based on the research reported by Tanaka et al. [20], the objective for the task-related component extraction is given by the Rayleigh–Ritz problem.

(1) $$\bar{\omega }=\arg\underset{\omega }{\max}\frac{\omega^{T}S\omega }{\omega^{T}Q\omega }$$

The normalization matrix Q is defined as:

(2) $$Q=\overset{N_{c}}{\underset{j_{1},j_{2}=1}{\sum }}Cov\left(x_{j_{1}}\left(t\right),x_{j_{2}}\left(t\right)\right)$$

where $x_{j_{1}}\left(t\right)$ is the EEG data in $j_{1}$-th channel and $x_{j_{2}}\left(t\right)$ is the EEG data in $j_{2}$-th channel. $N_{c}$ is the number of total channels. The $Cov\left(.,.\right)$ represents the cross covariance.

The symmetric matrix $S={\left(S_{j_{1}j_{2}}\right)}_{1\leq j_{1},j_{2}\leq N_{c}}$ is defined as:

(3) $$S_{j_{1}j_{2}}=\overset{N_{t}}{\underset{\begin{array}{c} h_{1},h_{2}=1\\ h_{1}\neq h_{2} \end{array}}{\sum }}Cov\left(x_{j_{1}}^{\left(h_{1}\right)}\left(t\right),x_{j_{2}}^{\left(h_{2}\right)}\left(t\right)\right)$$

where $x_{j_{1}}^{\left(h_{1}\right)}\left(t\right)$ is the EEG signal in $h_{1}$-th trial in the $j_{1}$-th channel. $x_{j_{2}}^{\left(h_{2}\right)}\left(t\right)$ is the EEG signal in $h_{2}$-th trial in the $j_{2}$-th channel.

With the help of the Rayleigh–Ritz theorem, the eigenvector of the matrix $Q^{-1}S$ provides the optimal coefficient vector of the objective function in (1). Finally, utilizing the EEG data $X_{i}=\left\{x_{j}^{h}\right\}\left(h=1,2,\ldots ,N_{t},j=1,2,\ldots ,N_{c}\right)$ (*i* is the index of stimulus), eigenvalues $\lambda_{1},\;\lambda_{2},\;\cdots ,\lambda_{N_{c}}$ and eigenvectors $\omega_{1},\omega_{2},\cdots ,\omega_{N_{c}}$ can be obtained for *i*-th stimulus. 

Unlike previous SSVEP studies, in which the largest eigenvalue was selected, the current study tested whether the components were significantly task-related or not and automatically selected all the significant task-related components to construct spatial filters.

#### 2.2.2. Chosen of Significant Components

The eigenvalues represent the task consistency among multiple trials. If the original signals contain no task-related component, the corresponding eigenvalues will be limited to a chance range. Thus, a non-parametric, permutation test [24] is introduced to assess the significance level of the task-related components in this study. The null hypothesis postulates that there is no task-related component. Instead of the actual EEG data $X_{i}=\{x^{\left(h\right)}\}\left(h=1,2,\ldots ,N_{t}\right)$ which is segmented by event timestamps, a new dataset $\chi_{i}$ is randomized to segmented $N_{t}$ times (sampled from a uniform distribution of entire experimental duration). This new dataset can be used to compute the null distribution of the weight distribution.

The statistical significance of actual coefficients can be quantified by comparing with the null distribution. The null hypothesis is rejected at a significance level 0.01. The eigenvalues $\lambda_{1},\;\lambda_{2},\;\cdots ,\lambda_{N_{s}}$ whose statistic is greater than 99% of the null distribution (*p_value*) can be regarded as being statistically significant.

Finally, $N_{e}$ significant eigenvalues are selected. Then, corresponding eigenvectors are chosen as the spatial filters. Since there are $N_{f}$ ($N_{f}=4$ in current study) different calibration data corresponding to the visual stimuli, $N_{f}$ different spatial filters corresponding to each eigenvalue can be integrated. Thus the final spatial filter $W_{1},\;W_{2},\;\cdots ,W_{N_{e}}$ can be described as follows.

(4) $$\left\{\begin{array}{c} W_{1}=\left[\omega_{11}\;\omega_{12}\;\cdots \;\omega_{1N_{f}}\right]\\ W_{2}=\left[\omega_{21}\;\omega_{22}\;\cdots \;\omega_{2N_{f}}\right]\\ \begin{array}{c} \vdots \\ W_{Ne}=\left[\omega_{Ne1}\;\omega_{Ne2}\;\cdots \;\omega_{NeN_{f}}\right] \end{array} \end{array}\right.$$

where $N_{f}$ is the number of stimuli. 

${\left(X_{i}\right)}^{T}W_{\kappa }\;\left(\kappa =1,2,\ldots ,\;N_{e}\right)$ are the significant task-related components. Through spatial filtering $X_{\mathrm{test}}{}^{T}W_{i}$, the test data $X_{test}$ is expected to be optimized to achieve maximum performance.

#### 2.2.3. Feature Extraction 

The correlation coefficient is selected as the feature. The Pearson’s correlation analysis between the single-trial test signal $X_{test}$ and average training data ${\bar{X}}_{i}$ across trials for *i*-th stimulus is calculated as:

(5) $$r_{\kappa }=\rho \left(X_{test}{}^{T}W_{\kappa },{\left({\bar{X}}_{i}\right)}^{T}W_{\kappa }\right),\kappa =1,2,\ldots ,\;N_{e}$$

where ρ is Pearson’s correlation analysis.

Considering the fact that the difference of the amplitude in spectrum among those significant eigenvectors is slight, the correlation coefficient $r_{\kappa \_TRCA}$ based on TRCA is calculated as follows.

(6) $$trca\_r_{i}=\frac{1}{N_{e}}\overset{N_{e}}{\underset{\kappa =1}{\sum }}r_{\kappa }$$

Finally, the target can be identified by the following equation.

(7) $$\iota =arg\underset{i}{\max}\left(trca\_r_{i}\right)\;,i=1,2,\ldots ,N_{f}$$

Since CCA have the ability to maximize the covariance between sine-cosine reference signals and EEG data, a combined mc-TRCA (CB-mc-TRCA) method was proposed. It combined CCA-based spatial filter and TRCA-based filter together. The correlation analysis based on CCA is calculated as:

(8) $$W_{i\_CCA}=canoncorr\left(X_{i},Y_{f_{i}}\right),Y_{f_{i}}=\left\{\sin f_{i},\cos f_{i}\right\}$$

where $f_{i}$ is the frame rate corresponding to the *i*-th stimulus.

(9) $$cca_{r}{}_{i}=\rho \left(X_{test}{}^{T}W_{\kappa_{CCA}},{\left({\bar{X}}_{i}\right)}^{T}W_{\kappa_{CCA}}\right),\;W_{\kappa \_CCA}=\left[W_{1\_CCA}\;W_{2\_CCA}\;\cdots \;W_{N_{f}\_CCA}\right]\;$$

The final features $com\_\rho_{i}$ are obtained by merging the correlation coefficients corresponding to CCA and TRCA, which are calculated by the following equation.

(10) $$com\_\rho_{i}=sign\left(cca\_r_{i}\right)\cdot {\left(cca\_r_{i}\right)}^{2}+sign\left(trca\_r_{i}\right)\cdot {\left(trca\_r_{i}\right)}^{2}\;,i=1,2,\ldots ,N_{f}$$

The target could also be identified by finding the maximal coefficient $com\_\rho_{i}$.

### 2.3. Ensemble Task-Related Component Analysis (e-TRCA)

Ensemble task-related component analysis (e-TRCA) [20] was first proposed in 2018 to deal with SSVEP data. It achieved better performance than existing CCA-based methods such as extended CCA [17]. E-TRCA utilized TRCA to construct the spatial filter. Considering $N_{f}$ different spatial filters obtained from eigenvectors corresponding to the largest eigenvalue in each stimulus, they should be similar to each other [25]. Thus, integrating all spatial filters might further improve the performance, compared to TRCA. Actually, the ensemble spatial filter is equal to $W_{1}=\left[\omega_{11}\;\omega_{12}\;\cdots \;\omega_{1N_{f}}\right]$ in the current study. The target was also identified by finding the maximal coefficient.

### 2.4. CCA-Based Method

CCA [15] is the most widely used method in SSVEP processing. Multi-channel EEG data and template signals are calculated by the following formula.

(11) $$\rho \left(x,y\right)=\frac{E\left[w_{x}^{T}XY^{T}w_{y}\right]}{\sqrt{E[w_{x}^{T}XX^{T}w_{y}E[w_{x}^{T}YY^{T}w_{y}]]}}$$

where *ρ* is the to CCA correlation coefficient, *X* is the EEG data, and *Y* is the template signals.

Our recent study [9] optimized the template signals specifically for the designed gaiting stimulus. The template signals *X* is described as follows.

(12) $$Y=\left\{\begin{array}{c} \begin{array}{c} \sin\left(2\times \pi \times Cb\left(i,1\right)\times t\right)\\ \cos\left(2\times \pi \times Cb\left(i,1\right)\times t\right) \end{array}\\ \sin\left(2\times \pi \times Cb\left(i,2\right)\times t\right)\\ \begin{array}{c} \cos\left(2\times \pi \times Cb\left(i,2\right)\times t\right)\\ \sin\left(2\times \pi \times Cb\left(i,3\right)\times t\right)\\ \cos\left(2\times \pi \times Cb\left(i,3\right)\times t\right) \end{array} \end{array}\right\},\;i=1,2,3,4$$

(13) $$Cb=\left[\begin{array}{c} \begin{array}{ccc} F_{1} & 2\times F_{1} & F_{1}+2\times f_{1} \end{array}\\ \begin{array}{ccc} F_{2} & F_{2}-2\times f_{2} & F_{2}+2\times f_{2} \end{array}\\ \begin{array}{c} \begin{array}{ccc} F_{3} & F_{3}-2\times f_{3} & 2\times F_{3} \end{array}\\ \begin{array}{ccc} F_{4} & F_{4}-2\times f_{4} & F_{4}+2\times f_{4} \end{array} \end{array} \end{array}\right]$$

where $F_{1}$= 8.57 Hz, $f_{1}$ = 0.536 Hz, $F_{2}$ = 12 Hz, $f_{2}$ = 0.75 Hz, $F_{3}$ = 10 Hz, $f_{3}$ = 0.625 Hz, $F_{4}$ = 15 Hz, $f_{4}$ = 0.938 Hz.

The target on which the participant focused on could also be identified by taking the maximum CCA coefficient. 

### 2.5. Cross-Validation

The offline analysis was done in MATLAB software. A 4-fold cross-validation scheme was performed for the EEG data. Of the four runs’ data for each participant, a single run was retained as the training data, and the remaining three runs were used as test data. There was no overlapping part in both training and test subsets. Then, the cross-validation process was repeated four times, with each four runs’ data used exactly once as the training data. The ratio of the number of correct classification to the total number was the accuracy. Finally, the average accuracy in different runs was used as the metric to qualify the performance.

### 2.6. Statistical Analysis

The mixed effect model of analysis of variance (ANOVA) was used for statistical analysis. Participant was used as the random factor. Data length (1, 1.5, 2, 2.5, 3) and method (“1”: CCA, ”2”: e-TRCA, “3”: mc-TRCA, “4”: CB-mc-TRCA) were used as fixed factors. Accuracy value was the response variable. The Bonferroni post hoc analysis was used to assess significance. The statistical significance level was 0.05.

## 3. Results

### 3.1. Analysis of Eigenvalues and the Significant Task-Related Components

**Figure 2:**
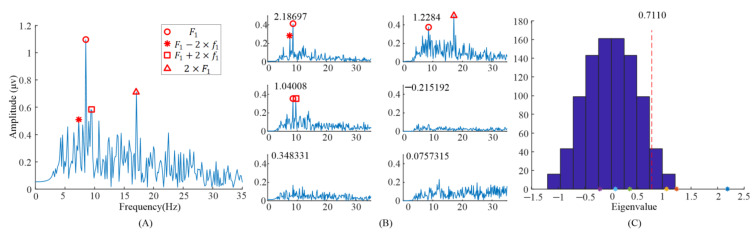
Analysis of the eigenvalues and task-related components in the EEG data when S6 gazed at the left gaiting stimulus. (**A**) The spectrum of the averaged EEG data. (**B**) The spectrums of task-related components. (**C**) The eigenvalue distribution using randomized task onsets. The asterisks (*) are the eigenvalues at the upper left of sub figure in (**B**).

The spectrum of the averaged EEG data, and the spectrums of task-related components and eigenvalue distributions using randomized task onsets from the EEG data of the subject 6 are illustrated in Figure 2. For the analysis of the task-related components, five blocks’ EEG data, when S6 observed the left stimulus ($F_{1}$= 8.57 Hz, $f_{1}$ = 0.536 Hz), were included. Figure 2A showed the spectrum of the entire 6 s EEG data during task period from Oz electrode. The EEG data were averaged across all the trials with the same task in the first run. The peaks in the spectrum could be clearly identified exactly at the frame rate ($F_{1}$) and the second harmonic frame rate ($2\times F_{1}$),while other peaks in the frequencies ($F_{1}\pm 2\times f_{1}$) reported in [9] were not clear.

When we applied TRCA based on Equation (1) to the EEG data, six eigenvalues and eigenvectors were obtained. Figure 2B illustrates the spectrums of task-related components (${\left(X_{i}\right)}^{T}W_{\kappa }$). The corresponding eigenvalues were at the upper left of the subgraph in Figure 2B. It can be clearly observed that the peaks in the spectrums with different eigenvalues were different. The peaks, in the spectrum with eigenvalue 2.18697, were at $F_{1}$ and $F_{1}-2\times f_{1}$. The peaks, in the spectrum with eigenvalue 1.2284, were at $F_{1}$ and $2\times F_{1}$. Finally, the peaks, in the spectrum with eigenvalue 1.04008, were at $F_{1}$ and $F_{1}+2\times f_{1}$. Figure 2C illustrates the eigenvalues’ distribution computed with randomized task onsets (blue bars) and original eigenvalues (asterisk). The vertical dashed line indicates the 99% confidence interval; there were three eigenvalues on the right side of the vertical dashed line. This indicated that these three components were statically significant, i.e., corresponding task-related components as described before were significant. 

**Figure 3:**
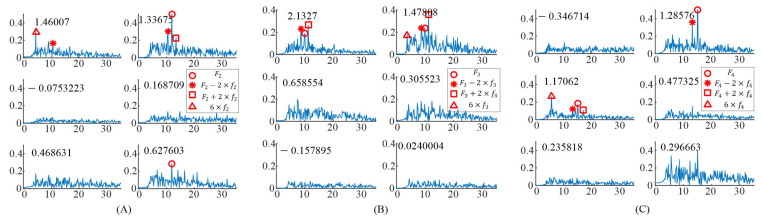
The spectrums of task–related components when S6 gazing at the different gaiting stimulus. (**A**) Gazing at the right gaiting stimulus. (**B**) Gazing at the up gaiting stimulus. (**C**) Gazing at the bottom gaiting stimulus.

For the EEG data responding to the right stimulus, the up stimulus, and the bottom stimulus, the spectrums of task-related components (${\left(X_{i}\right)}^{T}W_{\kappa }$) were obtained as shown in Figure 3. The induced frequencies were also distributed in different components. For the right stimulus target ($F_{2}$ = 12 Hz, $f_{2}$ = 0.75 Hz), there were three significant task-related components, as shown in Figure 3A. The peaks at $6\times f_{2}$ and $F_{2}-2\times f_{2}$ occurred in the spectrum of the first significant task-related component (corresponding to eigenvalue 1.46007). The peaks at $F_{2}$ and $F_{2}\pm 2\times f_{2}$ occurred in the spectrum of the second significant task-related component (corresponding to eigenvalue 1.33675). The peak at $F_{2}$ occurred in the spectrum of the third significant task-related component (corresponding to eigenvalue 0.627603). 

For the up stimulus target ($F_{3}$ = 10 Hz, $f_{3}$ = 0.625 Hz), there were two significant task-related components as shown in Figure 3B. In addition, the peaks at $F_{3}$ and $F_{3}\pm 2\times f_{3}$ occurred in the spectrum of the first significant task-related component (corresponding to eigenvalue 2.1327). The peaks at $F_{3}$, $6\times f_{3}$, and $F_{3}\pm 2\times f_{3}$ occurred in the spectrum of the second significant task-related component (corresponding to eigenvalue 1.47808). 

For the bottom stimulus target ($F_{4}$ = 15 Hz, $f_{4}$ = 0.938 Hz), there were two significant task-related components, as shown in Figure 3C. The peaks at $F_{4}$ and $F_{4}-2\times f_{4}$ occurred in the spectrum of the first significant task-related component (corresponding to eigenvalue 1.28576). The peaks at $F_{4}$, $6\times f_{4}$, and $F_{4}\pm 2\times f_{4}$ occurred in the spectrum of the second significant task-related component (corresponding to eigenvalue 1.17062).

In addition, Table 1 shows the distribution of features, i.e., modulated frequencies, induced by the gaiting stimuli in the significant task-related components in all participants. The number “1”, “2”, and “3” in the table represented the task-related component corresponding to the largest eigenvalue, the second largest eigenvalue, and the third largest eigenvalue, respectively. “-” represented that there was no peak at this frequency in the spectrums of all the significant task related components. In addition, the order of the numbers corresponded with the amplitude of the peaks at the modulated frequencies. First order meant higher amplitude. The result showed that the modulated frequencies occurred in different task-related components instead of in the component corresponding to the largest eigenvalue. Moreover, the amplitude of the peak at the modulated frequency in “1” was not always larger than that in “2”, e.g., $F_{2}$ in S3, $F_{1}$ in S4, $F_{3}$ in S5, and so on.

### 3.2. Target Identification Performance

**Figure 4:**
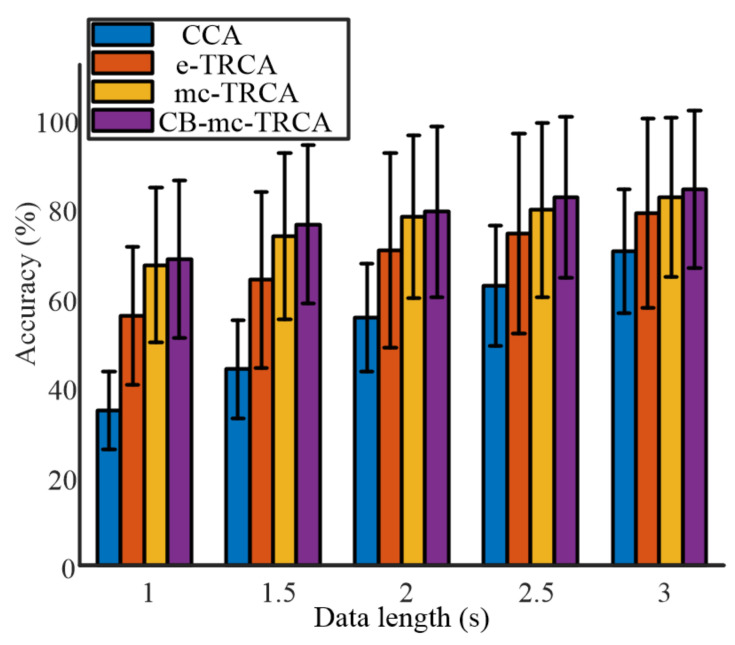
The comparison of accuracy among the four different algorithms with different data lengths.

Figure 4 shows the averaged classification accuracy across all the participants with different data lengths from 1 s to 3 s with an interval of 0.5 s. The comparison of the four methods indicated that the proposed mc-TRCA and CB-mc-TRCA methods outperformed the CCA-based method and e-TRCA method, especially at short data length. In addition, the CB-mc-TRCA achieved the highest performance regardless of data length. It is worth noting that the average accuracy with 1 s data length increased from 34.8% to 68.5%. Further, with the increase of the data length, the accuracy increased using all the methods. According to the ANOVA analysis, both the factor data length (*F* (4771) = 185.08, *p* < 0.001) and method (*F* (15,771) = 82.85, *p* < 0.001) had significant effects on accuracy. The post hoc comparison revealed that the accuracies using mc-TRCA and CB-mc-TRCA methods were significantly higher than the accuracies using CCA-based method within the data lengths 1 s, 1.5 s, 2 s, 2.5 s, and 3 s (all *p* < 0.001). In addition, the accuracies using mc-TRCA were significantly higher than the accuracies using e-TRCA with data lengths 1 s, 1.5 s, and 2 s (*p* < 0.001, *p* < 0.001, *p* = 0.007). The accuracies using CB-mc-TRCA were significantly higher than the accuracies using e-TRCA with data lengths 1 s, 1.5 s, 2 s, and 2.5 s (*p* < 0.001, *p* < 0.001, *p* < 0.001, *p* = 0.047). Even though the average accuracy using CB-mc-TRCA was higher than that using mc-TRCA, there was no significant difference regardless of data length (all *p* > 0.1).

Chiang et al. recently proposed a statistically optimized spatial filtering in decoding SSVEP based on TRCA. Utilizing Chiang’s method, the average identification accuracy achieved 61.13% ± 19.54%, 68.63% ± 21.39%, 74.46% ± 22.54%, 78.13% ± 22.44%, and 80.71% ± 20.92% with data lengths 1 s, 1.5 s, 2 s, 2.5 s, and 3 s, respectively. The accuracies were higher than the accuracies using CCA and e-TRCA methods but lower than the identification accuracy using the proposed mc-TRCA and CB-mc-TRCA methods.

**Figure 5:**
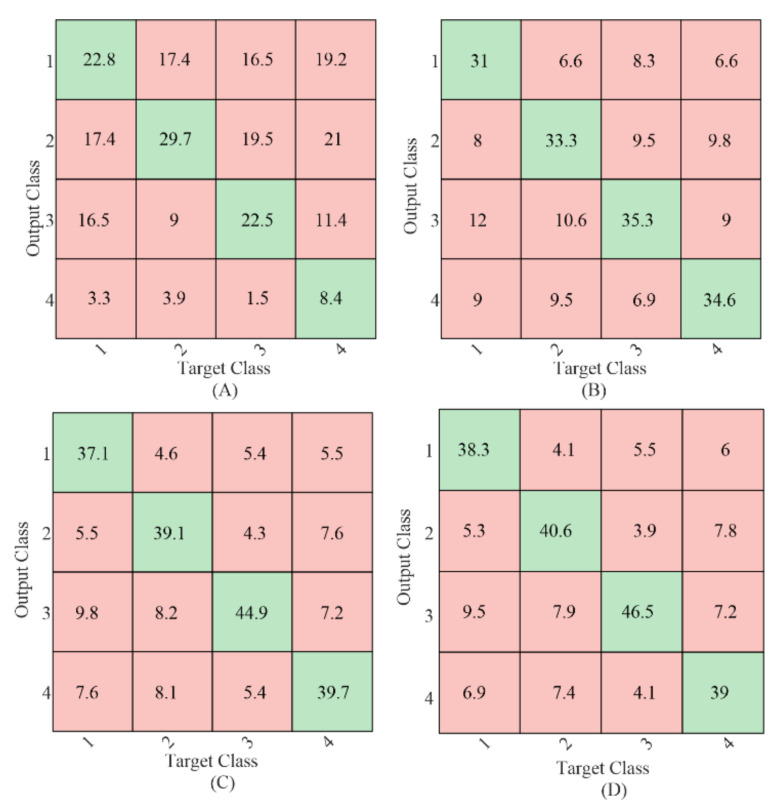
The classification confusion matrices average from all the subjects. (**A**) CCA-based method; (**B**) e-TRCA method; (**C**) mc-TRCA method; (**D**) CB-mc-TRCA method.

To further compare the relative target identification performance of the gaiting stimulus using different methods, the confusion matrices of the sum of the 4-fold cross-validation results with 1 s EEG data length averaged from all the subjects were calculated as shown in Figure 5. The values in the green background (diagonal entries) were the number of correct classifications, and the values in the red background (off-diagonal entries) were the number of misclassifications. Target 4 resulted in the fewest correct classifications using CCA-based method as shown in Figure 5A, which was in line with our previous study in [9]. No such difference was found for the classification using the TRCA-based method. Comparing the diagonal entries, the correct classification number in each target using the proposed mc-TRCA method was higher than that using CCA-based method and e-TRCA method. This indicated that the improvement of the performance was contributed to all the targets instead of one target.

**Figure 6:**
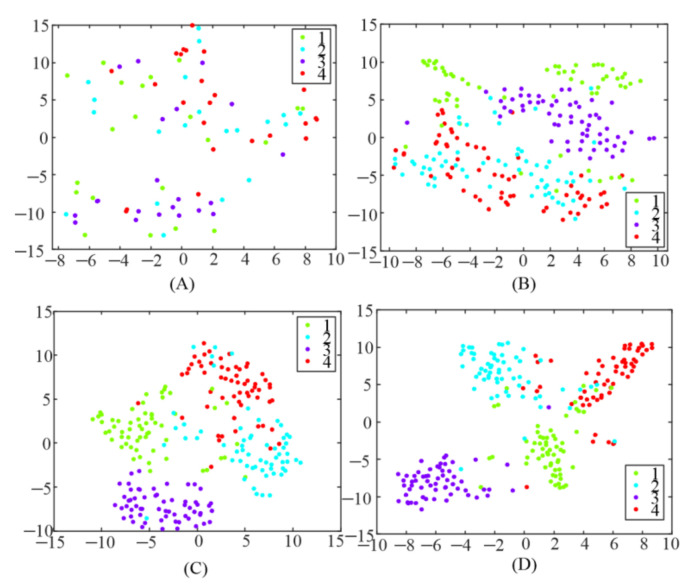
Feature visualization using t-SNE. (**A**) The correlation coefficient after utilizing CCA-based spatial filter; (**B**) the correlation coefficient after utilizing e-TRCA-based spatial filter; (**C**) the correlation coefficient after utilizing mc-TRCA-based spatial filter; (**D**) the correlation coefficient after utilizing CB-mc-TRCA-based spatial filter.

In addition, TRCA was utilized to generate a spatial filter to enhance the detection of SSMVEP in this study. To test the influence of different spatial filters, the extracted features, after spatial filters were applied, were visualized using a t-stochastic neighborhood embedding (t-SNE) [26] technique, as illustrated in Figure 6. Each point in the figure belonged to 1 s data length of a single trial and was colored based on the target label. It could be observed that the features were randomly distributed everywhere after applying CCA-based spatial filter as shown in Figure 6A. The data points became more clustered when an e-TRCA-based spatial filter was used, compared with the CCA-based spatial filter. However, there was still a certain number of features mixed together. When the proposed TRCA-based spatial filters (mc-TRCA and CB-mc-TRCA) was used, as shown in Figure 6C,D, it led to better clustering and class separation. Only a few data points clustered in the wrong category.

**Figure 7:**
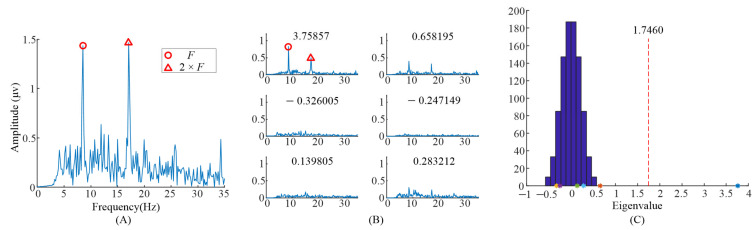
Analysis of the eigenvalues and task-related components in the EEG data when S6 gazed at the flicker stimulus. (**A**) The spectrum of the averaged EEG data; (**B**) the spectrums of task-related components; (**C**) the eigenvalue distribution using randomized task onsets.

**Figure 8:**
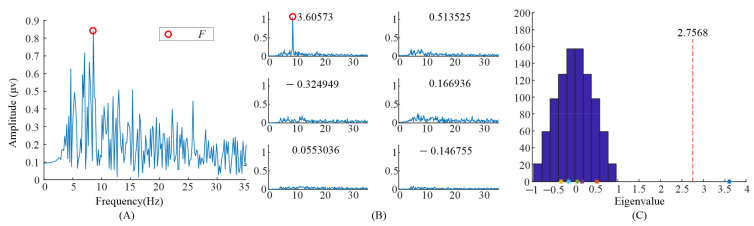
Analysis of the eigenvalues and task-related components in the EEG data when S6 gazing at the checkerboard stimulus. (**A**) the spectrum of the averaged EEG data; (**B**) the spectrums of task-related components; (**C**) the eigenvalue distribution using randomized task onsets.

Furthermore, the eigenvalues and task-related components in the EEG data when S6 gazed at the left flicker stimulus and checkerboard stimulus, as we demonstrated in our previous study [9], were also illustrated. As shown in Figure 7 and Figure 8, only one significant task-related component was found. Moreover the features, i.e., fundamental frequency and its second harmonic frequency, mainly occurred in the task-related component corresponding to the largest eigenvalue. This might be the reason why previous studies only selected the largest eigenvalue to construct the spatial filter.

## 4. Discussion

The results indicate that (1) the proposed TRCA-based methods are capable of automatically detecting several significant task-related components from the EEG data induced by the gaiting stimulus, and (2) provide a significant advantage with short duration of stimulation (≤2 s). Furthermore, CB-mc-TRCA showed a slightly better performance.

EEG induced by the flicker stimulus containing dual frequencies revealed the underlying nonlinearity [27,28,29]. A similar phenomenon occurred in the EEG induced by the motion stimulus with dual frequencies [21]. Even though the frequencies induced by different stimuli with dual frequencies are not the same, all the induced frequencies are among m×F±n×f. For the AO stimulus, our recent study reported the underlying nonlinearity response for the first time [9]. The main SSMVEP frequencies induced by the gaiting stimulus were at the frame rate, the sum and difference between the frame rate and twice stride frequency. However, the mechanism of inducing the modulated frequencies is still unknown. In the current study, the frequencies induced by the gaiting stimulus were further explored. The results in Section 3.1 revealed that the modulated frequencies occurred in the significant task-related components. These results indicated that the SSMVEP induced by the gaiting stimulus indeed appeared consistently and robustly in every task block and further proved the validity of the modulated frequencies induced by the gaiting stimulus. 

Beyond our expectation, several significant task-related components were obtained and the modulated frequencies distributed in different task-related components. Even though the characteristic of the distribution had not been obtained, the result still indicated that the induced frequencies in the EEG data were not simply linear superposition or completely coupled together. 

To our best knowledge, this is the first study applying TRCA to analysis the EEG data induced by AO stimulus. The results revealed that applying TRCA was able to construct the spatial filters (eigenvectors). The peaks at the different modulated frequencies were clearer after applying different spatial filters corresponding to different eigenvalues. When only the spatial filter corresponding to the largest eigenvalue is utilized, not all the induced modulated frequencies were selected as the features for classification. This might be the reason that utilizing all the significant task-related components could improve the detection performance compared with only utilizing the task-related component with the largest eigenvalue. Finally, the designed spatial filter could lead to a better cluster for the exacted features as shown in Figure 6. 

In view of the above, we speculate that the proposed mc-TRCA approach was feasible for detecting SSMVEP induced by other AO stimuli and enhancing the detection performance.

Furthermore, not only the stimulus frequency but also modulated frequencies were induced by the stimulus with multi-frequencies. Thus, the template signals utilizing CCA were difficult to manually select and optimize [12,21]. Yet mc-TRCA is a data-driven approach, so that there is no need to select optimal template signals. Moreover, the requirement for the size of the training data is not strict. Just as described in the current study, only twenty trials (eighty trials in total) were used as the training data and a good detection performance was achieved. 

The gaiting stimulus was designed for BCI-based rehabilitation. It was the first AO stimulus that depended on the characteristics induced by movement, instead of flicker [8], for classification. It guaranteed that the identified characteristics and the activation of the motor cortex came from the same source (i.e., the movement in the gaiting stimulus). Enhancing the detection performance might improve the effect of rehabilitation training. The results in the current study showed that the proposed TRCA-based method improved the identification accuracy, especially in short task duration, which might be more beneficial for BCI-based rehabilitation. increasing the task duration increases, the participants might be more likely to feel tired and the EEG data might be more likely to mix with noise. 

Currently, our approach focuses on offline detection of SSMVEP induced by gaiting stimulus. However, for practical applications, online detection performance needs to be assessed. One run of data is utilized as the training data and the significant task-related components could be automatically selected, hence making the proposed TRCA-based approach a suitable candidate for online AO-based BCIs. Thus, future work will be conducted to: 1) develop a real-time closed-loop AO-based BCI rehabilitation training system using the proposed TRCA-based approach, 2) collect and analyze the stroke patients’ EEG data when they gaze at the designed AO stimulus.

## 5. Conclusions

We conclude that (1) different modulated frequencies induced by the gaiting stimulus are distributed in different significant task-related components; (2) the proposed TRCA-based approaches are a suitable candidate for detecting SSMVEP induced by AO stimulus and can provide improved performance. In particular, we further demonstrate that the gaiting stimulus could induce modulation frequencies, and these frequencies could be utilized to perform classification. Moreover, the accuracies using the proposed methods were significant higher than that using CCA at all window lengths and significantly higher than those using ensemble-TRCA at short window lengths (≤2 s). This is especially true for the average accuracy with 1 s data length, which increased from 34.8% to 68.5%.

## Figures and Tables

**Table 1:** The distribution of the modulated frequencies induced by the gaiting stimuli in the significant task-related components among all participants.

Targets	Features	S1	S2	S3	S4	S5	S6	S7	S8	S9	S10
Left target	$F_{1}$	1,2	1	1	2,1	1,2	1,2,3	1,2,3	1,2	1,3	3,1
	$2\times F_{1}$	1,2	1	1	-	1	2	2	-	1	-
	$F_{1}+2\times f_{1}$	-	1	1	2	-	3	2,3	2	2	2
	$F_{1}-2\times f_{1}$	2	1	2,3	1	-	1	1,2,3	1,2	3	3
Right target	$F_{2}$	1,2	1	2,1	1	1	2	1,2	1	2,1	1
	$6\times f_{2}$	1,2	1	1,2	2,1	1	1	2	1	-	1,2
	$F_{2}+2\times f_{2}$	1	1	2	1	1,2	2	2	1	-	-
	$F_{2}-2\times f_{2}$	1	1	2,1	1	1,2	2,1	1	1	-	1
Up target	$F_{3}$	1,2	1	2,1	1	2,1	2,1	1	1	2,1	2
	$6\times f_{3}$	2	-	1	2	1	2	2,1	1	1	1
	$F_{3}+2\times f_{3}$	1,2	1	-	1	1	2,1	2	1	1	2
	$F_{3}-2\times f_{3}$	1,2	1	2,1	1	2	2,1	2,1,3	-	1	1
Bottom target	$F_{4}$	2,1	1	1,2	-	2,3	1,2	1,2,3	2,1	1,2	1
	$6\times f_{4}$	1,2	2,1	2,1	3	1	2	1,2	1,2	2	-
	$F_{4}+2\times f_{4}$	1	1	1	1	-	2	-	-	1	-
	$F_{4}-2\times f_{4}$	2,1	1,2	2,1	2	2	1,2	1,3,2	1	2,1	-

Note: “1”, “2”, “3” represented the task-related component corresponding to the largest eigenvalue, the second largest eigenvalue, and the third largest eigenvalue, respectively. “-” represented there was no peak at this frequency in the spectrums of all the significant task related components.

## Data Availability

No new data were created or analyzed in this study. Data sharing is not applicable to this article.

## Supplementary Materials

The following are available online at https://www.mdpi.com/article/10.3390/s21165269/s1, Figure S1: Generation of the gaiting stimulus, Figure S2: Illustration of the experiment protocol.
