# Supplementary figures and images for: Enhancing Detection of SSMVEP Induced by Action Observation Stimuli Based on Task-Related Component Analysis
Source: Sensors (Basel). 2021 Aug 4;21(16):5269. doi: 10.3390/s21165269 (PMC8400839; doi:10.3390/s21165269)

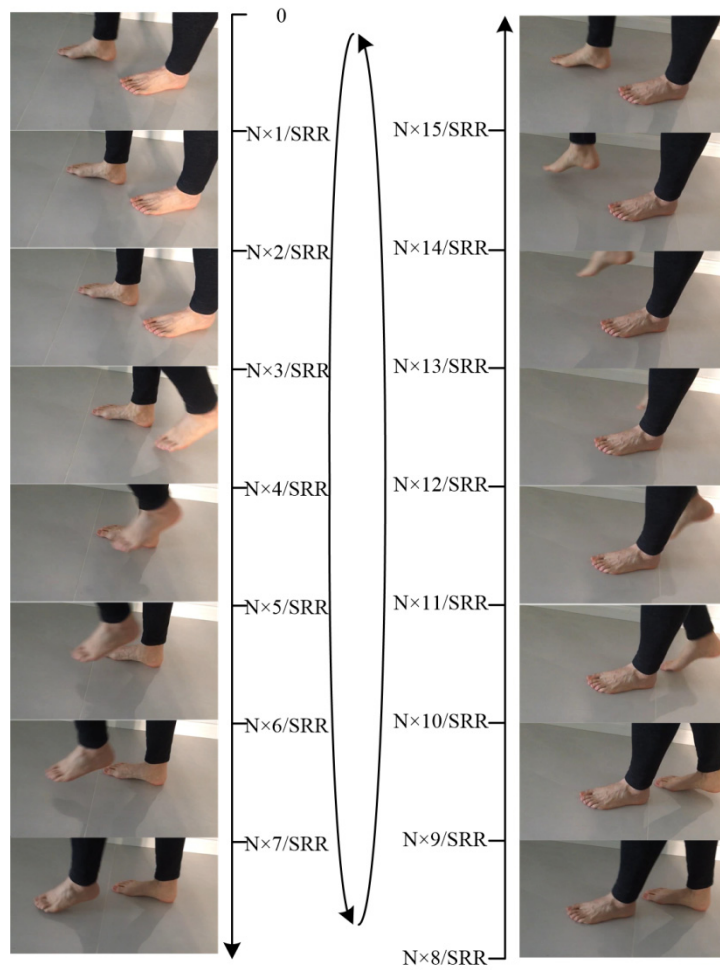

**Figure S1.** Generation of the gaiting stimulus.

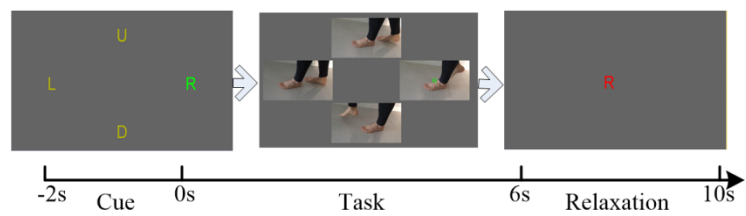

**Figure S2.** Illustration of the experiment protocol.

Supplement: Supplementary file 1 [file sensors-21-05269-s001.zip › sensors-1291483-supplementary.pdf]
